# Sentience, Vulcans, and zombies: the value of phenomenal consciousness

**DOI:** 10.1007/s00146-023-01835-6

**Published:** 2024-01-12

**Authors:** Joshua Shepherd

**Affiliations:** 1 Universitat Autónoma de Barcelona, Facultat Filosofia y Letres, Carrer de la Fortuna, Cerdanyola del Vallès, 08193 Barcelona, Spain; 2 ICREA, Passeig Lluís Companys 23, Barcelona, Spain

**Keywords:** Phenomenal consciousness, moral status, AI sentience, zombies

## Abstract

Many think that a specific aspect of phenomenal consciousness—valenced or affective experience—is essential to consciousness’s moral significance (valence sentientism). They hold that valenced experience is necessary for well-being, or moral status, or psychological intrinsic value (or all three). Some think that phenomenal consciousness generally is necessary for non-derivative moral significance (broad sentientism). Few think that consciousness is unnecessary for moral significance (non-necessitarianism). In this paper, I consider the prospects for these views. I first consider the prospects for valence sentientism in light of Vulcans, beings who are conscious but without affect or valence of any sort. I think Vulcans pressure us to accept broad sentientism. But I argue that a consideration of explanations for broad sentientism opens up possible explanations for non-necessitarianism about the moral significance of consciousness. That is, once one leans away from valence sentientism because of Vulcans, one should feel pressure to accept a view on which consciousness is not necessary for well-being, moral status, or psychological intrinsic value.

## Introduction

The view that phenomenal consciousness is morally significant is widely held across philosophy (Kriegel 2019), and variants of this view are influential in discussions of moral status (Harman 2003; Shepherd 2018; DeGrazia 2021) well-being (van der Deijl 2021; Lin 2021), and intrinsic value (Lee 2019), as well as in more focused discussions of phenomena like treatment of those with traumatic brain injury (Kahane and Savulescu 2009, Weijer et al. 2014), regulation of research using brain organoids (Koplin and Savulescu 2019; Sawai et al. 2021), and the moral status of artificial intelligence (AI) and robot rights (Torrance 2008; Véliz 2021) as well as machine ethics (Nath and Sahu 2020). But a general agreement that consciousness is in some way morally significant masks substantive disagreement regarding the specific nature of this significance.

Call *valence sentientism* regarding some normative property P, the view that only sentient beings instantiate P-valenced phenomenally conscious experiences are necessary, and on most versions of the view sufficient, for instantiating P. Thus understood, valence sentientism is popular throughout value theory, and many take it to be at least a part of the right view regarding moral status (e.g., Shepherd 2018; DeGrazia 2021; Gibert and Martin 2022), well-being (Crisp 2006; Bramble 2016; Lin 2021), and the distribution of intrinsic value (Lee 2019).^1^

A *broad sentientist* view regarding P maintains that, while valenced experiences may be sufficient for instantiating P, phenomenal consciousness generally is both necessary and sufficient. Only conscious beings—beings who either have tokened or have the capacity to token^2^ mental states with phenomenal properties, beings for whom there is ‘something it is like’ to token mental states—instantiate the normatively relevant property or properties. One finds defense of this view in Chalmers (2022) and useful discussion in Roelofs (2023), as we will see below.

A third view is that, while phenomenal consciousness in some form may be sufficient for moral significance, it is not necessary. Other aspects of mentality may also be sufficient for moral significance. Let us call this view *non-necessitarianism*.

What is at stake in the debate between valence sentientism, broad sentientism, and non-necessitarianism? The theoretical issue of intrinsic interest is the (non-derivative) moral significance of consciousness. And there are important practical implications as well. In this paper I consider some strange beings—Vulcans, angels, zombies, human-like robots. These may seem esoteric, but progress in artificial intelligence may one day place us into contact with many types of being, some in virtual worlds, some in our own, that resemble Vulcans or, for all we know, zombies (Butlin et al. 2023; Schwitzgebel 2023). And progress in stem cell technology is rapidly advancing, bringing us closer to synthetic biocomputational intelligences constructed out of, for example, neural cells embedded into silicon computational architectures (Kagan et al. 2022; Smirnova et al. 2023). Sooner than we realize, our views about the moral significance of consciousness, or about aspects of mentality separable from consciousness, may become very practically important, highlighting only some beings as deserving of moral or legal protections.

In this paper I first consider the prospects for sentientism and broad sentientism in light of Vulcans, beings who are conscious but without affect of any sort. But the discussion uncovers sources of theoretical pressure in a novel direction—one that rejects the claim that phenomenal consciousness is necessary for attributing moral significance (that is, well-being, moral status, or psychological intrinsic value) to someone. My aim here is thus twofold. First, I aim to clarify and flesh out the options for sentientists, broad sentientists, and non-necessitarians. Second, I aim to articulate sources of theoretical pressure that I think have gone under-appreciated in recent discussions of these positions, it may surprisingly push some of us to accept the moral significance of zombie mentality.^3^

## Vulcans and angels

Broad sentientism is less popular than valence sentientism. But an intuition in its favor can be elicited by the case of Vulcans—a case that Chalmers has recently offered as a part of an argument against sentientism (which he calls narrow sentientism).

Let’s say that a *Vulcan* is a conscious creature who experiences no happiness, suffering, pleasure, pain, or any other positive or negative affective states . . . Vulcans’ lives may be literally joyless . . . But they may nevertheless have serious intellectual and moral goals. They may want to advance science, for example, and to help those around them. They might even want to build a family or make money. They experience no pleasure when anticipating or achieving these goals, but they value and pursue the goals all the same. (Chalmers 2022, 417–419)

Do Vulcan mental states bear non-derivative value? I propose a quick walk through a range of answers, because I wish to better understand what our options are regarding the value of phenomenal consciousness and the viability of various versions of sentientism. Let us begin with defensive reactions on the part of the valence sentientist.

The valence sentientist might say, initially, that Vulcan mental states do not bear non-derivative value, because the Vulcan case is defective, because Vulcans are inconceivable, or impossible. In actual fact, any level of cognitive sophistication, such as the one Vulcans are said to possess, will be connected to valenced experiences. These need not be experiences of sensory pleasure and pain, of course. But they will be valenced nonetheless—experiences like a sense of mental effort, a sense of curiosity or interest, or a sense of processing fluency.^4^

Although I think that human cognition is shot through with valenced experience, this response is difficult to sustain in general. It requires there to be a priori links between cognitive processes, cognitive states like desires, preferences, and expectations, and valenced experiences. One can make the case for this idea, but one faces an uphill climb. For we seem to have little trouble talking about non-conscious desires and preferences, and their satisfaction.

The more direct sentientist response is that the Vulcan case does not present us with a plausible example of moral status, or well-being of any level,^5^ or psychological intrinsic value. This seems to be DeGrazia’s response when he considers a case similar to Vulcans.

Imagine angels who are conscious but, lacking feelings, not sentient, and who have the aim of performing certain actions simply because they are right. Even if they do not feel good upon achieving their aims or bad if their aims are thwarted, they have interests in noninterference . . . (DeGrazia 2021, 43)

DeGrazia rejects the thought that, in virtue of these interests, the angels have moral status. His reason is that ‘The possession of values or aims the fulfillment of which one does not care about (emotionally) *at all*—if the terms ‘values’ and aims’ are even apt in such a case—seems insufficient for having anything at stake, any interests or welfare’ (43).^6^

I think DeGrazia’s line here is a good one for the committed valence sentientist to take, and I confess I find it somewhat compelling. The angels, like the Vulcans, might be thought of as no different, morally, than a super-computer capable of solving difficult mathematical, logistical, or even moral problems. An interesting type of cognitive machine, but not one we should worry about turning off.

Broad sentientists offer an opposed judgment. They judge that the psychological life of the Vulcan, or an angel, instantiates some normative property. Chalmers, for example, claims that killing the Vulcan is obviously morally wrong, even monstrous: ‘It doesn’t matter that the Vulcan has no happiness or suffering in its future. It’s a conscious creature with a rich conscious life. It cannot be morally dismissed in the way that we might dismiss a zombie or a rock’ (2022 419).^7^

I confess I find this judgment somewhat compelling as well. The harder I stare at Vulcans or angels, the less sure I am about valence sentientism. We might (and I do) find the prospect of a switch from human to Vulcan chilling. But I find it difficult to rule out the existence of value in the psychological life of a Vulcan.

So I, at least, am confronted with a tension. It may help, then, to see what reasons each side may offer in their favor, beyond judgments about cases.

## Explanations for valence sentientism

One thing the valence sentientist has regarding the experiences at the root of their view is a handy explanation. The valence sentientist can say that valenced experiences are valuable *because* the experiences are good or bad. Valenced experiences wear their value on their sleeve, so to speak—the value is there in the phenomenal character, in what it is like to have the experiences.

The value of valenced experiences may thus ground the attribution of welfare, moral status, or intrinsic value to the experiencer. A nearby valence sentientist explanation of moral significance appeals to the way that valenced experiences ground interests. According to the well-known resonance constraint,^8^ what is valuable in a person’s psychological life should have some connection to what they find valuable or attractive. So Railton, for example, writes,

[I]t does seem to me to capture an important feature of the concept of intrinsic value to say that what is intrinsically valuable for a person must have a connection with what he would find in some degree compelling or attractive, at least if he were rational and aware. It would be an intolerably alienated conception of someone’s good to imagine that it might fail in any such way to engage him. (Railton 1986, 47)

The idea in play here is that valenced experiences are what ground the attribution of morally significant interests to the experiencer. These are interests that in turn ground the moral status of the experiencer, or her status as a welfare subject.

The same sort of explanations are not as readily available regarding non-valenced experiences. They feel neither good nor bad. And they arguably do not ground interests in the same way. As DeGrazia indicates, for the valence sentientist, there seems to be nothing *at stake* for the subject with respect to non-valenced experiences.

## Explanations for broad sentientism

What explanations can the broad sentientist offer for the claim that non-valenced experiences are (non-derivatively) morally significant?

Option one is a relatively brute appeal to intuition. Just as valenced experiences are intrinsically valuable, say, the broad sentientist may press the line that non-valenced experiences are intrinsically valuable. One worry with this claim surfaces when we consider cases of simpler experiences, as Andrew Lee has done.

Consider two worlds that are empty save for a single creature inhabiting each world. In the first world, the creature has a maximally simple conscious experience that lacks any valence. Perhaps, for example, the creature has an experience of slight brightness. The creature’s experience is exhausted by this sparse phenomenology. In the second world, the creature is not conscious at all. (Lee 2019, 663)

Lee and others (myself included) judge that neither world contains non-derivative value. Suppose one accepts that judgment, but judges nonetheless that the Vulcan’s psychological life is non-derivatively morally significant. How might the broad sentientist explain the difference between the judgments?

Recall that when explaining the claim that it would be wrong to kill a Vulcan, Chalmers claimed that the Vulcan is ‘a conscious creature with a rich conscious life’ (419). Building upon this, the broad sentientist might posit a spectrum of phenomenal richness, and claim that non-derivative value (or moral status, or well-being) only enters into the picture once an entity’s psychological life meets a certain level or threshold of richness.

To make this option appealing, we need to see what kind of explanation is available regarding this threshold idea. What differentiates the non-derivatively valuable level of phenomenal richness from the non-valuable level? At this point, a worry enters in, for one might think that all we are doing is adding non-valuable experience types to a psychological subject’s repertoire. We begin with the experience of slight brightness, say, then we add different aspects of vision, then audition and tactile experiences, various bits of cognitive phenomenology, and so on and on. It is not clear why we should think that adding a few more non-valuable experience types will turn the heap into an intrinsically valuable heap.

Possibly, the intuition about Vulcans is undergirded by the thought that the richness of their mental life involves cognitive sophistication. Many views about well-being and moral status, at least, either appeal to cognitive sophistication or directly appeal to it.^9^ But one has to be careful here. Non-conscious beings, like zombies, appear to possess cognitive sophistication. And on many understandings of cognitive sophistication, AIs do—or will soon—clear the bar (for relevant discussion, see Andreotta 2021). Short of further explanation of the kind of cognitive sophistication in view, the broad sentientist risks opening themselves to an error theory—an argument that the broad sentientist has confused the value of cognitive sophistication for the value of a bundle of non-valenced experiences. This kind of issue returns below.

A second way to understand phenomenal richness is in esthetic terms. Certainly some conscious experiences—the experience of self, the experience of free will, certain vivid or intense perceptual experiences, cognitive experiences associated with contemplation of paradoxes, or the sublime—have a phenomenology one might describe with a range of normatively loaded descriptors. We might think of certain experiences as interesting, fascinating, beautiful, deeply meaningful, and more. Of course, important aspects of many of these experiences do not survive if we remove all valence or affective charge. But experiences may still be beautiful, even without the positive affect associated with interesting or beautiful phenomenology. Rich experiences may be thought to be a part of the class of intrinsically valuable experiences.

But again, it is not clear that non-valenced, rich experiences are on their own sufficient for moral significance. We can try to imagine a mind composed of entirely passive, perceptual experiences. Perhaps a cerebral organoid coaxed into forming an analog of the visual cortex, along with optic cups to permit stimulation via light (Eiraku et al. 2011). We can imagine a wide array of vivid, intense, varied perceptual experiences pass through this mind. It seems that such a mind might be interesting as a kind of object of esthetic significance (though none of us could appreciate the esthetic properties of its experiences). But it does not seem that this passive perceptual mind qualifies as a welfare subject, or as an entity with moral status. It has no interests—or at least, whatever interests we might ascribe to it are similar to what we might ascribe to a plant. If we reject intrinsic or non-derivative value in Lee’s case of slight brightness, there is pressure to reject non-derivative value here as well.^10^ So it is not at all clear that rich, unvalenced experiences are on their own sufficient for moral significance.

A different kind of option is offered by Jonathan Birch.

What the philosophical Vulcan shows us, I suggest, is that morally significant interests can be grounded independently of valence. An autonomous rational being capable of reflectively endorsing goals and projects has such interests, whether or not it has experiences of frustration, joy (and so on) associated with the success or failure of those projects. Note, however, that the Vulcan is still registering the promotion or frustration of its interests in experience. I propose that the step up in moral status associated with phenomenal consciousness is the change that comes when events that promote or thwart a being’s interests are registered in experience. (Birch 2022, 800)

According to Birch, for the Vulcan it is the *conscious* registration of interests that matters. What is unclear, at this point, is why this registration needs to be phenomenally conscious. On this option the interests, without valence, are not valuable. It is not terribly plausible that a general experience of registration of some fact—registering that the sun is setting, for example—is valuable. There is little reason to think that the registration of some random mental fact (registration that this is an imaginative episode, as opposed to a memory, for example) will be valuable. Why, then, does the conscious registration of interests generate value?

One answer is inspired by Moore’s view on organic wholes. Moore (1903) thought that while conscious experiences were of some value, and objective goods such as a beautiful thing were of some value, much more valuable were the objective wholes composed of consciousness and its (good, or beautiful) object. One option then is that, while interests on their own are not morally significant, and while conscious mental acts of registration are not independently morally significant, the conjunction of conscious registration with an agent’s interests is a morally significant whole. I do not find this very plausible, for a simple reason. Zombies are capable of registering interests.

A different way of interpreting the registration idea gives consciousness a unique role to play in the registration. Some think that consciousness provides a special kind of access to, or contact or acquaintance with, items in the world or in the agent’s own mind. This kind of contact is why Shepherd (2018) maintains that independently of valence, consciousness is necessary (though not sufficient) for non-derivative value. Consciousness—in the sense of the most determinable phenomenal property, ‘what-it-is-like-ness’—is constitutive of what Shepherd calls presence:

[O]f all the events that constitute a subject’s mental life, those events presented to her within consciousness are special. Those events are present to her. As Bertrand Russell might have put it, they are *before her mind* in a certain way. Furthermore, there is an important relationship between presence and there being something it is like. In short, the property of what-it-is-like-ness that an item of conscious experience essentially possesses is constitutive of the presence of that item before an agent’s mind. (Shepherd 2018, 37)

For Shepherd, some mental state is non-derivatively valuable if and only if it contains affective aspects, and in addition it is conscious, thus ‘secur[ing] presence to the subject’ (37).

Whatever the plausibility of that view, a different version is available to the broad sentientist. The broad sentientist may maintain that what-it-is-likeness secures presence to the subject of various items (or, if you like, the subject’s ‘acquaintance’ with available items), and either that [a] this mode of access is, on its own, non-derivatively valuable, or [b] this mode of access, when conjoined with items of objective (dis)value, grounds (dis)value for the subject.

Regarding option [a], certainly many philosophers have found acquaintance—a kind of conscious mental relation to some set of items that provides direct contact with items in the set (see Raleigh 2019; Duncan 2021)—a special phenomenon.^11^ But the specialness has been thought to be primarily a matter of epistemology, and secondarily one of metaphysics. A large project (beyond the present scope, as they say) might build bridges into value theory. However, there is an initial reason to doubt that acquaintance, on its own, provides the sort of moral significance after which we are asking. The reason is the wide range of experiences that seem neither valuable nor disvaluable, which seem unimportant for moral theory. Lee’s experience of slight brightness is one example. The slight breeze I feel now on my arm, or the straightness of my back, or my memory of walking to this café minutes ago, are others. These cases suggest that acquaintance needs, at least, to be paired with certain items, before it becomes morally relevant.

So we move to option [b]. I think there is something attractive about this option. Acquaintance is said to provide a kind of direct contact with items. If those items are valuable, then the subject will be in direct contact with these valuable items—a relation that seems, itself, valuable.

One hurdle for this claim in the context of Vulcans revolves around the sorts of items one can confront via acquaintance. Many have thought that what acquaintance provides is contact with items within one’s experience (Giustina 2022). Duncan explains:

You might think that even if I met the actor in person—saw him, shook his hand, talked to him, etc.—that would be different from the way I am aware of my headache. You might think, and more than a few philosophers have thought, that I am only indirectly aware of the actor in virtue of being aware of my experiences—my sensations—of him. If that is right, then my awareness of my experiences—such as my headache—really is different from my awareness of other things. (Duncan 2021, e12727)

Not everyone thinks this, of course—at one time Russell did not (see Raleigh 2019). It becomes an important choice point in the context of Vulcans. For Vulcans do not have available to them the wide range of valenced experiences uncontroversially regarded as non-derivatively valuable. Vulcans may still have some non-derivatively valuable experiences available to them—what might they be? Intuitions will probably vary. The examples that seem compelling to me are few and far between. A sense of selflessness achieved during intense meditation. The agentive experiences associated with the perfect execution of some action (minus the usual positive valence). Bizarre perceptual phenomena associated with hallucinogenics. The relevant issue here is not getting the list of experiences right. It is the rarity of the experiences on the list. These may be enough to generate some moral significance (and so, to falsify valence sentientism), but they may not be enough to explain the judgment that the Vulcan, for example, has the same moral status as a human, or that the Vulcan’s mental life contains a similar level of non-derivative value as a human’s.

Those who share these judgments may argue that acquaintance directly connects subjects with more than their experiences. Some philosophers have argued for this view (e.g., Bonjour 2003; Duncan 2015). But it is not clear that these arguments help with our current predicament. Duncan (2015), drawing on Russell and others, offers the following doubt test for determining items with which we can be acquainted.

**The doubt test**: If it seems to S that she is aware of some x, and on the basis of that seeming awareness S cannot doubt (psychologically) that x exists, and S can rule out all skeptical scenarios in which x does not exist, then S is acquainted with x. (Duncan 2015, 2541)

Duncan argues that the self passes muster. But one might argue that few experience external items, especially items of value, uncontroversially do so. Consider what kind of items might be required. Many mundane items (the beauty of a sunset, someone’s display of courage, a friend’s gracious gesture) seem ruled out simply because one can doubt the presence of features essential to them (the sunset, one’s friend). Additional problems surround whether one can have experiences of, e.g., beauty, without any affectively valenced experience. One might think more abstract features are still in play. Some have thought, for example, that we can be acquainted with universals. One might thus argue that Vulcans could be acquainted with some moral facts.

Or consider that objective list theories of well-being often cite items as goods that contribute to well-being, even when those goods seem to have little essential connection to (affective) consciousness. Candidates include desire satisfaction, perfection of one’s nature, development of one’s capacities, self-respect, achievement, knowledge, and the development of friendships (see Fletcher (2015) for an overview). How many of these pass the doubt test?

Though more work is required, and welcome, I submit that none of these items uncontroversially pass the doubt test.^12^ There are good reasons that philosophers who discuss acquaintance focus on sensory experiences.

However, in spite of the difficulties (and the undone work) associated with this acquaintance option, it seems the best option available to the broad sentientist. Note, in this connection, that broad sentientism is only a claim about the necessity of conscious experience. On the acquaintance explanation, the broad sentientist could argue that acquaintance is necessary, and additional cognitive capacities are also necessary, to give birth to non-derivative value, or morally significant interests, in a psychological entity.

If consciousness provides acquaintance with some items of value, then, this may be enough to ground the broad sentientist’s claim that consciousness itself is necessary to explain the moral significance of Vulcan mentality. There is space here for growth. For it seems that there are argumentative routes, at least, toward the claims that Vulcans can be acquainted with some non-valenced but non-derivatively valuable experiences that Vulcans can be acquainted with the self, and perhaps some moral facts or universals.

## An explanation for non-necessitarianism

I have argued that an appeal to acquaintance with items of value is the best option for explaining the appeal of Vulcan cases. I want now to consider the implications for non-necessitarianism—the claim that consciousness is not necessary for the ascription of moral significance to some entity’s mental life.

Many recent writers reject non-necessitarianism, with most regarding it as a non-starter. An exception is Kagan (2019), who offers the following case.

Imagine that you are an Earth scientist, eager to learn more about the makeup of these robots. So you capture a small one—very much against its protests—and you are about to cut it open to examine its insides, when another robot, its mother, comes racing up to you, desperately pleading with you to leave it alone. She begs you not to kill it, mixing angry assertions that you have no right to treat her child as though it were a mere thing, with emotional pleas to let it go before you harm it any further.

Would it be wrong to dissect the child? (2019, 28).

Kagan offers a non-necessitarian judgment: ‘I find that I have no doubt whatsoever that it would be wrong to kill (or, if you prefer, to destroy) the child in a case like this. It simply doesn’t matter to me that the child and its mother are “mere” robots, lacking in sentience... For you to destroy such a machine really would be morally horrendous’ (28).

In response, Kriegel (forthcoming) offers the opposite judgment:

No matter how many experiential terms the vignette is surreptitiously peppered with (“desperately,” “angry,” “emotional”), and how many automatized projections it counts on from what such behavior in conscious beings indicates about their likely experiential state, one would have to be seriously confused to think that one is in any way harming a collection of metal plates by intervening in the metal’s internal organization (forthcoming).

When cases generate sharply conflicting judgments across a set of very sharp philosophers, it can be difficult to know how to proceed. My tack so far has been to ask what explanations we might offer for views that plausibly lie behind the judgments in question. Doing so, we arrived at what I think is the best explanation for broad sentientism. It appeals to acquaintance with valuable items. When thinking about the prospects for non-necessitarianism, one might be drawn to the following line of reasoning.

Acquaintance is one mode of cognitive contact with items of value. There are others. Consider that zombies, or Kagan’s robots, seem capable of maintaining friendships, appreciating art, or pursuing knowledge for its own sake. Zombies and robots are able to non-consciously contemplate philosophical paradoxes, and to non-consciously recognize the sublime or the beautiful. Indeed, although zombies do all of this non-consciously, they can retain awareness and knowledge of the mental states related to all of the above. When you ask a zombie what it is doing, they can answer, ‘I’m enjoying the sunset over that mountain ridge.’ And when you ask them why, they can answer, ‘Sunsets are beautiful. They distract me from my mundane concerns, and connect me with something bigger than myself.’

In many such cases, I want to say that the zombie pursuit of value is explained in part by the fact that zombies can have cognitive contact with items of value. What sort of contact? I want to say that provided by knowledge of the value that these items have (where ‘items’ is liberal, and includes the pursuit of valuable ends). It is plausible that, if anyone can, zombies can know that a painting is beautiful, that self-sacrifice is noble, that some kinds of life are worth living.^13^

Not everyone will agree.^14^ Smithies (2012) argues that there can be no cognitive zombie: cognition is essentially tied to consciousness. The argument runs through rationality. Cognition requires states that play rational roles, and the only states that can play rational roles are conscious or are individuated by their relations to consciousness. Why think that consciousness and rationality are thus connected? Smithies says the connection is fundamental. But he attempts to provide some sense of the connection by appealing to the practice of critical reflection:

The concept of rationality is essentially tied to the practice of critical reflection. To a first approximation, a belief is rational if and only if it is based in such a way that it would survive an idealized process of critical reflection. (2012, 364)

According to Smithies, an intentional state plays a rational role only if it is available via introspection. And introspection, for Smithies, requires consciousness.

Smithies’s perspective (see also Smithies 2019) thus offers one way to resist the claim that zombies have cognitive contact with items of value. I am not convinced, for two reasons. First, although in humans introspection involves consciousness by definition, when the consciousness of another entity is in question, the possibility of introspection without consciousness does not seem outlandish. It is possible to offer a functional account of introspection (see Morales forthcoming), and to ask whether a zombie or a sophisticated AI is capable of instantiating or approximating a capacity that works like our functional account says introspection works. The answer is yes. One might then ask whether this capacity is genuinely introspection, of course. The issue here may depend upon explanatory methodology regarding mental ontology. I am of the mind that if a capacity functions like our best science says a human capacity functions, and enters into similar explanations of behaviors (e.g., reports about mental contents), then it makes sense to attribute that capacity to a system, even if the consciousness of that system is in question.

Second, although the issue is too complicated to treat in detail here, I think we have good evidence that humans have unconscious states (like beliefs and intentions) that play rational psychological roles independent of introspection, supporting unconscious inferences (Quilty-Dunn and Mandelbaum 2018, unconscious adjustments to other beliefs (Porot and Mandelbaum 2021), and unconscious but rational adjustments to bodily and mental action (Shepherd and Mylopoulos 2021). This is not to deny important roles for conscious states in the normal operations of human rationality, of course. But if consciousness and rationality come apart in these ways in humans, then states that are neither conscious nor individuated by their relations to consciousness can play rational roles. This severs the essential connection between consciousness and rationality, and takes away a reason to rule out cognitive zombies.

What we need to ask, now, is whether a zombie who knows and pursues the valuable deserves any moral consideration in virtue of this fact—whether their interests matter morally, for their own sake. I am very tempted by a positive answer.^15^ I find it compelling to think that a psychologically sophisticated being capable of knowing things about what is valuable and capable of forming plans in light of this deserves the chance to work towards such knowledge and such plans. Notice that, as it should, this claim applies to Vulcans as much as zombies, and it is consistent with the thought that the psychological states of either being are not intrinsically valuable, and with the thought that neither are welfare subjects. For the ascription of moral status, then, the idea is that acquaintance is not special.^16^ What is important is cognitive contact. Acquaintance is one such mode. Knowledge had by other means—testimony, inference, imagination, whatever—provides others.

If one accepts that Vulcan mentality has moral significance because Vulcans can be acquainted with items of value, then one should feel some pressure to accept the claim that zombie mentality has moral significance because zombies are capable of knowledge of items of value. This is especially true for those who doubt the epistemic specialness of acquaintance, as well as for those who restrict the range of items with which consciousness provides acquaintance. For the way to resist this position regarding zombie moral significance is to show that acquaintance makes a moral difference that other modes of cognitive contact do not. I encourage broad sentientists to make such a case.

## An alternative explanation for non-necessitarianism

One might fairly complain that I have not identified the most plausible explanation for non-necessitarianism. This involves, not cognitive contact with items of value per se, but the structure that underlies such contact, namely, sufficiently sophisticated agency. Consider Kagan’s perspective on his non-conscious robots: ‘What matters... is that they are full-blown *agents*, with plans and hopes for their own lives, desires and ambitions for the future’ (2019, 28). Let us grant, going forward, that this is an explanatory option for the non-necessity view. Interestingly, if one is attracted to this view, one might leverage it to put pressure on broad sentientism.

To see how, note that Chalmers’s Vulcans and DeGrazia’s angels also seem to involve agency in important ways. It is important for Chalmers (2022) that Vulcans possess, value, and pursue serious intellectual and moral goals. DeGrazia (2021) describes angels in terms of an aim—the aim of performing certain actions simply because they are right. But if the broad sentientist wishes to appeal to agency as a part of the grounds of a Vulcan’s moral significance, then the broad sentientist needs to argue either [a] that agency necessarily involves consciousness, or [b] that while consciousness on its own is not morally significant, consciousness plus agency is, and agency without consciousness is not.

Option [b] would take some creativity to develop. As it stands, it strikes me as ad hoc. So I put it aside for now. Option [a] may have some legs, but if one thinks that zombies are conceivable, then one runs into immediate trouble here. For zombies seem to be agents as much as humans. Zombies have plans, intentions, goals, and they act intentionally.

Here, it may help to turn to a recent paper on Vulcans, where Roelofs (2023) explicitly appeals to agency to justify an intuition that Vulcans have moral significance.

For me at least, the force of the Vulcan argument comes from seeing the P-Vulcans as agents like me, striving to pursue their interests, able to ask for, receive, and extend empathy. They have conscious perspectives and value things from within those perspectives. (315)

Roelofs offers the following argument as a way of explaining Vulcan moral status.

Premise 1: an entity has moral status if and only if it has morally significant interests.

Premise 2: an entity's interests must be able to motivate it (resonance constraint).

Premise 3: phenomenal consciousness is a necessary condition for the sort of motivation involved in having interests. (basic sentientist commitment)

Conclusion: Therefore, an entity has moral status if and only if it is capable of undergoing motivating conscious states. (Motivational Sentientism) (2023, 317)

The key premise, for our purposes, is Premise 3. This premise connects phenomenal consciousness and agency via a claim about motivation. Roelofs offers it as a middle ground between two views on how conscious affect is related to the possession of morally significant interests. One view makes conscious affect necessary. A second view—the broad sentientist view—does not require conscious affect. Here is how Roelofs, referencing Chalmers, explains the view.

Chalmers glosses ‘motivating consciousness’ as ‘including affective conscious states but also (non-affective) conscious desires and judgments about value’ (Chalmers 2022). That is, it covers both affective consciousness, in which things feel nice or nasty, and also the sort of dispassionate motivating states that move P-Vulcans. Motivational Sentientism does not ascribe moral status to P-zombies, but does ascribe moral status to P-Vulcans, to affect-driven creatures like us, and to everything in between. But it denies moral status to conscious beings whose consciousness is not motivating. (315)

But—and this is the key question—why we think phenomenal consciousness is necessary for the non-affective sorts of desires and judgments about value that Vulcans possess? Short of an account here, it seems that zombies have as much claim to morally significant motivation as do Vulcans. So it seems that premise 3 is false.

Roelofs might respond that zombies are deluded in a way that undermines this conclusion. He writes that ‘Empathy with zombies is entirely possible, but it is by definition mistaken. We could ask a zombie what they care about, and get a sensible-sounding verbal response. And we could, based on that response or on observing the zombie's behavior, try to sympathetically imagine the zombie's perspective, and perhaps feel empathic motivation to help them. But by definition, the zombie's response is misleading, because zombies claim to feel things they do not feel…’ (312) But the response here is that we need only imagine zombified Vulcans (Z-Vulcans) who are not deluded, and could appeal to our empathy in a way similar to the Vulcan, asking us to appreciate, not their conscious experience, but their desires, goals, values, and plans. The question is whether we would owe a Vulcan, but not a Z-Vulcan, empathy. I cannot find a good reason to think so.^17^

The upshot is that the non-necessitarian appears to have an explanation—the appeal to agency—that the broad sentientist lacks.

## Conclusion

Let us take stock.

Valence sentientism looks very plausible when we reflect upon what is valuable about human mental life. Much that is valuable to us is inextricably entwined not only with consciousness, but with affective or evaluative aspects of conscious experience. In human beings, this is arguably as true of richly cognitive mental episodes as of sensory pleasures and pains. But stranger cases, like that of Vulcans, divorce affective elements of experience from consciousness itself. These cases place pressure on valence sentientism. It is possible for the valence sentientist to hold the line, of course, insisting that the best explanation for the value of consciousness resides in the goodness or badness of phenomenal character, and that without this all we find are beings for whom nothing really matters. But those who recoil at the imagined mistreatment of Vulcans or angels might wish to broaden their view to include non-valenced aspects of consciousness.

Making this move raises new theoretical pressures. Cases of simplistic, non-affective conscious experience suggest that phenomenal consciousness, on its own, is insufficient to ground important normative properties (like well-being, moral status, or intrinsic value). We have considered a few options for explaining the value of more sophisticated conscious experiences. In my view the best candidate leans on the way that consciousness provides acquaintance with items of objective value or disvalue.

This candidate explanation faces serious challenges, as we have seen. It also exposes broad sentientism to pressure from the view that phenomenal consciousness is not necessary for well-being, or moral status, or intrinsic psychological value. Consciousness may provide one mode of access to (or contact with) valuable items. But, if consciousness can be divorced from psychological sophistication in the way that robot and zombie cases suggest, then robots or zombies have a mode of access to valuable items as well. A being who only contacts such things via consciousness may insist that without consciousness, the contact is incomplete, or only virtual. It is within the zombie’s rights to insist that the conscious being does not know what they are talking about.

Further, the proponent of zombie moral significance has an explanation available that the broad sentientist lacks. The proponent of zombie moral significance can appeal to sophisticated agency to ground moral significance. This gives the non-necessitarian a dialectical leg up. The broad sentientist may wish to respond by developing the thought that consciousness is essential to sophisticated agency. That strikes me as a difficult task.

None of this is decisive, of course. Indeed, one thing that is difficult about the dialectics that this paper covers is that different positions depend upon intuitions about cases that seem to conflict across different philosophers, and that seem to wobble when confronted with different contexts or new thought experiments.^18^ I think conflict between, and instability regarding, intuitions at the heart of key judgments supporting a philosophical view should lead us to lower our credences overall, to explore our options carefully, and to pay attention to things like possible debunking explanations of our intuitions, and to the overall explanatory merits of a given view. Motivated by this kind of thought, my aim here has been in part to chart lines of thought in support of these different views on the moral significance of consciousness, and thereby to identify areas that require theoretical development. I think there is important work here to do in support of broad sentientism, especially regarding the scope of acquaintance, as well as the value-theoretic relevance of connections between acquaintance, cognitive phenomenology, and cognitive capacities.

From where we currently stand, I find that none of the three views I have considered compel unwavering support. I also think that, pending further reflection and argumentation, all three are viable candidates for reasonable assent. This may be most surprising regarding non-necessitarianism.

## Data Availability

There is no data associated with this article.

## Footnotes

1 A quick word: the way these three are related is open to further theorizing. Some would tie them closely together by, for example, making intrinsic value a part of the grounds of moral status, or even of well-being. But I put that aside for the purposes of this paper.

2 I will not worry here about the difference between exercised and unexercised capacities, though philosophers differ on this. See Harman (2003) for some discussion.

3 Not many have pushed in this direction explicitly. Earlier discussions include Carruthers (1999) and Levy (2014); recent discussions include Kammerer (2022), Bradford (2022), and Shepherd (2023).

4 Roelofs (2023) reflects usefully on this question, and notes that some views of the connection between consciousness and motivational states would render Vulcans impossible. Ultimately, Roelofs is uncertain whether Vulcans are possible, because they find such views ‘neither obviously correct nor obviously false’ (314).

5 Possibly one could say that the Vulcan possesses a ‘neutral good’ (ven der Deijl 2021) and as such qualifies as a welfare subject without any level of well-being (see also Lee 2022).

6 One might distinguish here between moral status and welfare subjecthood, arguing that angels have moral status in that they seem to possess some rights against interference, even if they lack welfare subjecthood. The question, then, is what grounds those rights. Possibly the agency or autonomy of the angels could do so, even in the absence of sentience. If one thinks that, then one will likely be sympathetic to the conclusion of this paper.

7 Zombies are non-conscious imagined beings that are physically or functionally identical with humans. There is a large literature surrounding the question of whether zombies are actually conceivable. I am granting for the present purposes that zombies are conceivable, although I doubt it.

8 In comments, [REMOVED] noted that it might be possible to construe the resonance constraint in terms of pro-attitudes toward the good (see Bradford 2022)—attitudes Vulcans could plausibly token. If so, one could argue that the appeal to the resonance constraint actually helps explain broad sentientism.

9 A family of views about moral status are sometimes called sophisticated cognitive capacities views: see Jaworska and Tannenbaum (2023).

10 Alternatively, one might argue that since a world with such minds is better than a world devoid of consciousness, the intrinsic value of consciousness is separable from moral status, or welfare subjecthood.

11 It is also a controversial notion. It is controversial just how much acquaintance can be used to explain, and whether the knowledge that acquaintance purportedly provides (or constitutes) is in some sense fundamental. It is controversial whether acquaintance can be reduced into some sort of physical-functional relation (Papineau 2016, Pautz 2017). It is controversial just how much direct contact with the world acquaintance provides—with just what sorts of things we can be acquainted (Atiq 2021). It is controversial whether acquaintance is a non-representational relation.

12 It might help here to develop the line of thought that cognitive phenomenology provides one acquaintance with certain abstract contents that seem non-derivatively valuable. See Levine (2011) for some thoughts in this direction.

13 Some may think that even if zombies are capable of knowing and pursing the good, this will not qualify them as welfare subjects. For a defense of experientialism about welfare subjecthood, see van der Deijl (2019). For a defense of the view that aspects beyond conscious experience qualify as welfare goods, see Bradford (2022). For a defense of this in the context of moral status, see Shepherd (2023).

14 Thanks to a referee for pressing this issue.

15 This would not, however, settle issues about relative degrees of moral status, or welfare.

16 It is important that this is *for the purposes of assessing moral status*. Nothing need be implied about the epistemic or metaphysical specialness of acquaintance.

17 Chalmers writes, ‘Philosophical zombies lack consciousness, so... there is arguably no one home to mistreat’ (2022, 415). This ‘no one home’ intuition may be at the root of broad sentientism’s appeal. It seems to me that it needs to be drawn out and given more explicit defense. It also strikes me as false. It seems that zombies will have values, personalities, life ambitions, and all the rest. Someone is home, even if the subjective life of that one is different or difficult to imagine.

18 Thanks to a referee for raising this issue.
